# Age and sex differences in sympathetic and hemodynamic responses to hypoxia and cold pressor test

**DOI:** 10.14814/phy2.13988

**Published:** 2019-01-18

**Authors:** Amanda J. Miller, Jian Cui, J. Carter Luck, Lawrence I. Sinoway, Matthew D. Muller

**Affiliations:** ^1^ Penn State Heart and Vascular Institute Penn State University College of Medicine Hershey Pennsylvania

**Keywords:** Blood pressure, femoral blood flow, microneurography, sex differences, sympathetic nervous system, vascular resistance

## Abstract

Emerging evidence suggests that sympathetic vasoconstriction is lower in young women. We hypothesized that increases in muscle sympathetic nerve activity (MSNA) during acute physiological stressors induce less vasoconstriction in young women compared to young men. Healthy young men (*n* = 10, 27 ± 1 years), young women (*n* = 12, 25 ± 1 years), and older women (*n* = 10, 63 ± 6 years) performed the cold pressor test (hand in ice for 2 min) and continuous hypoxia (10% O_2_, 90% N_2_) for 5 min. MSNA, femoral blood flow velocity, heart rate, and blood pressure were acquired continuously. Femoral artery diameter was obtained every minute and used to calculate femoral blood flow, and femoral vascular resistance and conductance. MSNA responses to cold pressor test (*P* = 0.345) and hypoxia (*P* = 0.969) were not different between groups. Young women had greater femoral blood flow (*P* = 0.002) and vascular conductance (*P* = 0.041) responses to cold pressor test compared with young men. The femoral blood flow response to hypoxia was not different between the two sexes but the increase in femoral flow was attenuated in older women compared with younger women (*P* = 0.036). These data show that young women had paradoxical vasodilation to cold pressor test. The mechanisms responsible for the attenuated sympathetic vasoconstriction or for enhanced vasodilation in young women during the CPT require further investigation.

## Introduction

Young women are protected from heart disease and are more prone to orthostatic hypotension than men are. Sex differences in the regulation of blood flow and vascular resistance may partially account for these clinical outcomes. The sympathetic nervous system is one mechanism to regulate peripheral vascular resistance by increasing alpha‐adrenergic receptor mediated vasoconstriction. Young women have lower resting muscle sympathetic nerve activity (MSNA) than men and while MSNA is correlated with peripheral resistance in men, it is not associated with peripheral resistance in women (Hart et al. 2009). However, the manner in which men and women respond differently to physiological stressors is less well understood. Previous studies show that young women have increased vasodilation to orthostatic (Shoemaker et al. 2001; Yang et al. 2012) and mental stress (Yang et al. 2013) as well as attenuated vasoconstriction to acute hypoxic stress (Patel et al. 2014). However, few of these studies measured arterial blood flow and MSNA simultaneously. Therefore, sex differences in arterial blood flow responses to physiological stressors that increase sympathetic activity have not been fully explained.

In this study we investigated the hemodynamic and neural responses to physiological stressors in young women, young men, and older women. We used two physiological stressors, the cold pressure test (CPT) and continuous hypoxia. Both of these tests increase MSNA (Victor et al. 1987; Rowell et al. 1989). CPT typically evokes alpha‐adrenergic receptor mediated peripheral vasoconstriction (Frank and Raja 1994) while hypoxia induces metabolic mediated vasodilation (Leuenberger et al. 1991). We hypothesized that young women have attenuated vasoconstriction to CPT and enhanced vasodilation to continuous hypoxia compared to young men and older women despite similar increases in MSNA.

## Methods

### Subjects and design

Thirty‐two healthy humans participated in this study. We recruited healthy young men and young women 18–30 years, and healthy older women 55 years and over. All subjects were nonsmokers, free of any systemic or chronic illness, and not taking medications other than hormonal contraceptives. The older women were all postmenopausal and none were on hormone replacement therapy. The young women were all premenopausal and 8/12 were on hormonal contraceptives (seven subjects took combination estrogen and progesterone pills and one had a progesterone only intrauterine device). Of the four subjects not on contraceptives, one subject did not have regular cycles, two were in follicular phase, and one was in luteal phase when studied based on self‐report. Because of the small sample sizes in each cycle phase, we were not powered to examine the effect of cycle phase on the physiological responses to hypoxia or CPT.

These experiments utilized a between subjects design in which physiological parameters and responses were compared between three groups. The sample size was determined after the first four subjects in each group had completed the study. We determined that if the true difference in the femoral blood flow (FBF) response to hypoxia between young men and young women was 27 mL/min with a standard deviation of 14, then we would need to enroll seven subjects for this variable to have 90% power and *α* = 0.05. Since it is not feasible to obtain successful recordings of MSNA in all subjects, we enrolled at least 10 subjects in each group to account for potential missing data. Each participant completed one study visit. If successful recordings of MSNA were not obtained at their first visit, participants were invited back for another visit 4 weeks later. Only two subjects participated in two visits and data from their first visits were not included in analysis.

### Experimental protocol

All study protocols were approved in advance by the Institutional Review Board of Penn State Milton S. Hershey Medical Center and conformed to the Declaration of Helsinki. All participants provided written and informed consent. The study protocols were performed in the supine position in a thermoneutral laboratory (20–21°C). After 15 min of quiet rest, baseline brachial artery blood pressures were measured in triplicate. Then subjects underwent CPT which involved placing the right hand in ice water for 2 min. This test was chosen since it raises MSNA and increases vasoconstriction (Victor et al. 1987; Frank and Raja 1994). Following recovery and at least 15 min of quiet rest, subjects were instrumented with a face mask connected to a gas monitor (Ohmeda RGM 5200, GE Healthcare) to record arterial oxygen saturation and End‐tidal CO_2_. Baseline brachial artery blood pressure was measured in triplicate and baseline MSNA and femoral blood flow velocity was recorded. Then subjects breathed room air for 3 min with the face mask on and then were switched to poikilocapncic hypoxia (10% O_2_) for 5 min. Continuous hypoxia was chosen since it increases MSNA but also evokes metabolic vasodilation (Leuenberger et al. 1991). Hypoxia was always performed last since this stressor can have sustained effects on MSNA (Leuenberger et al. 2005). At the end of the 5 min of continuous hypoxia, subjects performed a voluntary end‐expiratory apnea to further increase MSNA and test the validity of our MSNA recording at the end of the study (Greaney et al. 2010).

### Measurements

Subjects were instrumented with a 3‐lead EKG (Cardiocap, GE) to measure HR, a Finometer to measure beat‐to‐beat changes in BP (FMS, Amsterdam, The Netherlands), and a brachial artery oscilometric BP cuff (Phillips Sure Signs V3). The Finometer values were adjusted to the baseline BP values measured by the brachial artery cuff as previously described (Muller et al. 2014). Femoral artery velocity was measured continuously using duplex ultrasound. Images of the femoral artery were acquired every 30 sec; and femoral artery diameter was measured offline. Femoral artery blood flow (FBF; mL/min) was calculated as velocity (cm/sec) × area (2π*r* ^2^) × 60. Multifiber recordings of MSNA were measured using peroneal nerve microneurography as previously described (Leuenberger et al. 2005). Briefly, a tungsten microelectrode was inserted into the peroneal nerve at the popliteal fossa. A reference electrode was place 2–3 cm away from the recording electrode. The recording electrode was adjusted until a site with clear sympathetic nerve traffic to skeletal muscle was identified using established criteria (White et al. 2015). The nerve signal was amplified, band‐pass filtered with a bandwidth of 500–5000 Hz, and integrated with a time constant of 0.1 sec (662C‐3 Nerve Traffic Analysis System, University of Iowa Bioengineering, Iowa City, IA). The nerve signal was also routed to a loudspeaker and a computer for monitoring throughout the study. The integrated MSNA signal was evaluated offline using custom software to identify bursts based on fixed criteria of shape, latency following the R‐wave of the ECG, and 3:1 signal‐to‐noise ratio compared with the baseline.

### Data collection and statistical analysis

MSNA, BP, HR, and femoral artery velocity were measured continuously. Arterial O_2_ saturation and CO_2_ pressure were measured continuously during the hypoxia trial. Femoral blood flow was measured from the velocity and diameter obtained from the common femoral artery proximal to the bifurcation into superficial and deep femoral arteries. Two‐dimensional photos of the femoral artery were acquired every 30 sec and diameter was measured offline. Femoral blood flow (mL/min) was calculated as femoral artery velocity (cm/sec) × area × 60 sec/min. Femoral vascular resistance was calculated as mean BP/femoral blood flow and femoral vascular conductance was calculated as femoral blood flow/mean BP. All data were analyzed in 1 min bins. Baseline values were calculated as the average of 3 min prior to each stressor. Responses to CPT were calculated as the second minute of CPT minus baseline. Responses to hypoxia were calculated as the last minute of hypoxia minus baseline. Statistical analysis was performed using GraphPad Prism 7. Differences in baseline data and responses to physiological stressors were compared between groups using the Kruskal–Wallis test, which is a nonparametric one‐way analysis of variance on ranks (ANOVA). For significant *P‐*values, post‐hoc Dunn's multiple comparisons tests were performed. Significance was set at *P* < 0.05 for all tests. Data are shown as mean ± SEM.

## Results

### Anthropometric and baseline data

Anthropometric and baseline data are shown in Table 1. Data on age, height, and weight in the three study groups are shown in Table 1. Diastolic BP was higher in older women compared with both younger groups (*P* < 0.0001). There were no differences in heart rate between groups (*P* = 0.7758). Baseline MSNA was higher in older women compared with both young men and women (*P* < 0.0010) with no differences between young groups (*P *=* *0.8844). There are no significant differences in baseline femoral blood flow between groups but femoral artery diameter was smaller in young women compared to young men (*P* = 0.0248).

**Table 1:** Anthropometric and Baseline Data

	Young men (*n* = 10)	Young women (*n* = 12)	Older women (*n* = 10)	*P‐*values
Age (years)	27 ± 1	25 ± 1	63 ± 6^a^, ^b^	<0.001
Height (m)	1.82 ± 0.02^b^	1.62 ± 0.02^a^	1.51 ± 0.16 a	<0.001
Weight (kg)	85.3 ± 2.4^b^	60.2 ± 1.6^a^	63.6 ± 7.2^a^, ^b^	<0.001
BMI (kg/m^2^)	25.7 ± 0.61	22.8 ± 0.38	23.2 ± 2.8	0.079
Systolic BP (mmHg)	114 ± 3	105 ± 2	123 ± 2^b^	<0.001
Diastolic BP (mmHg)	65 ± 2	67 ± 2	78 ± 1^a^, ^b^	<0.001
Heart Rate (beats/min)	56 ± 3	59 ± 2	57 ± 1	0.776
MSNA (beats/min)	9 ± 1 (*n* = 8)	7 ± 0 (*n* = 7)	21 ± 4^a^, ^b^ (*n* = 7)	<0.001
Femoral blood flow (mL/min)	137.1 ± 30.3	106.8 ± 10.6	81.0 ± 15.1	0.187
Femoral diameter (cm)	0.64 ± 0.03	0.54 ± 0.03^a^	0.56 ± 0.01	0.026

Baseline data are taken from the last 3 min of a 15 min period of quiet rest prior to the Cold Pressor Test trial.

a *P* < 0.05 compared to young men.

b *P* < 0.05 compared to young women.

### Responses to the cold pressor test

**Figure 1:**
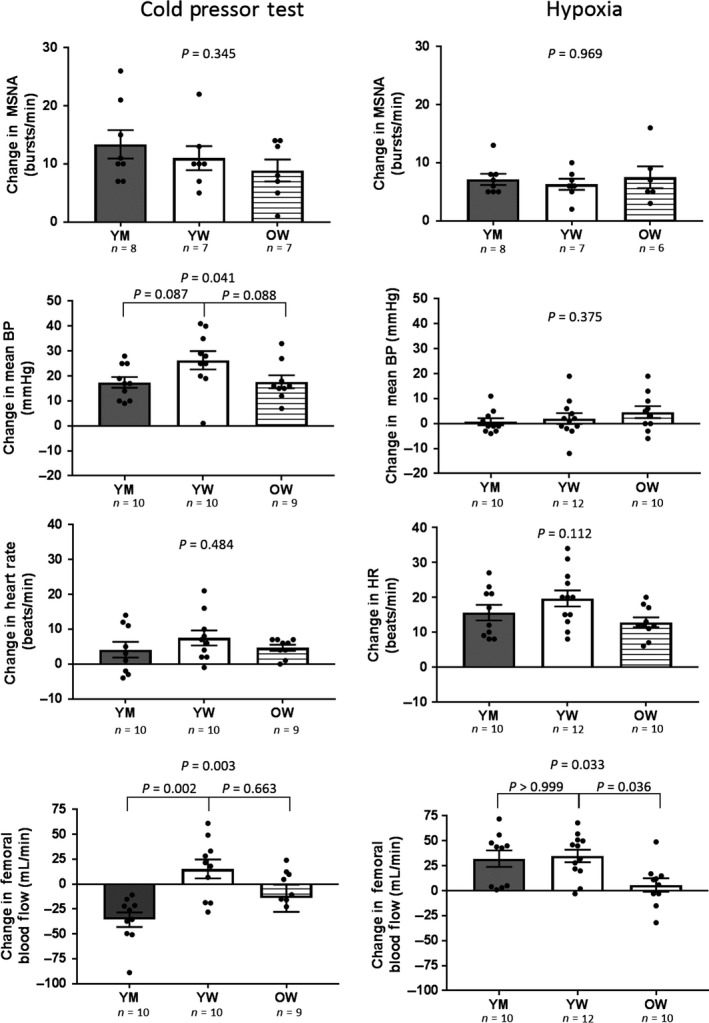
Muscle sympathetic nerve activity (MSNA), mean blood pressure (BP), heart rate (HR), and femoral blood flow responses to 5 min of continuous hypoxia and the cold pressor test. Data are shown as changes from baseline (last minute of stressor minus baseline) in each group. Bar graphs show group mean ± SEM; dots represent individual subjects. Between group analysis of variance were performed using Kruskal‐Wallis test with Dunn's multiple comparisons. Young men, YM; young women, YW; older women, OW.

**Figure 2:**
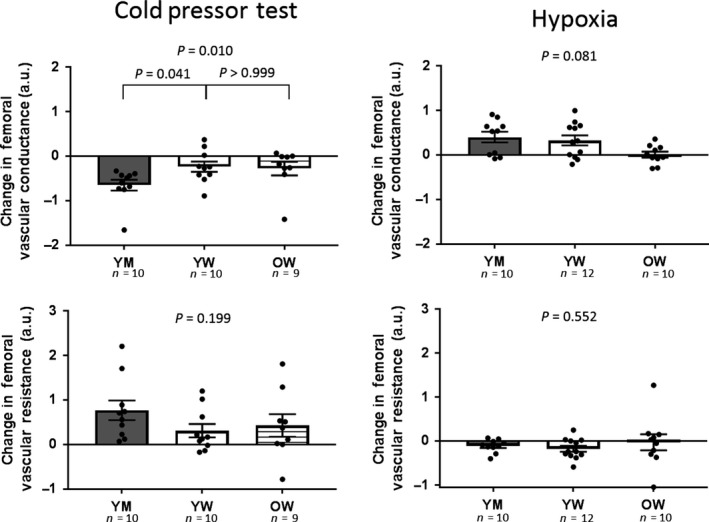
Femoral artery vascular conductance and resistance responses to 5 min of continuous hypoxia and the cold pressor test. Data are shown as changes from baseline (last minute of stressor minus baseline) in each group. Bar graphs show group mean ± SEM; dots represent individual subjects. Between group analysis of variance were performed using Kruskal–Wallis test with Dunn's multiple comparisons. Young men, YM; young women, YW; older women, OW.

Two young women and one older woman did not tolerate CPT and their data during CPT were not analyzed. CPT was completed by 10 young men, 10 young women, and nine older women. Physiological responses to CPT are shown in Figure 1. MSNA recordings were obtained during CPT in eight young men, seven young women, and seven older women. Physiological responses to CPT are shown in Figure 1. MSNA responses to CPT were not different between groups (*P* = 0.3449). There was a trend toward higher mean BP response to CPT in young women compared with older women (*P* = 0.0869) and to young men (*P* = 0.0884) but neither comparison was significant. CPT did not significantly affect HR (<10% change in each group) and there were no differences between groups (*P* = 0.4843). While CPT decreased femoral blood flow in young men and older women, most young women had an increase in femoral blood flow in response to CPT. The femoral blood flow response to CPT was greater in young women compared to young men (*P* = 0.0090) and tended to be greater in young women compared to older women but this did not reach statistical significance (*P* = 0.1176). Changes in femoral vascular conductance and resistance are shown in Figure 2. Young women had an attenuated decrease in femoral vascular conductance with CPT compared to young men (*P* = 0.0406). But, changes in conductance were not different in young and older women (*P* > 0.9999). Femoral vascular resistance responses to CPT were not different between groups (*P* = 0.1993).

### Responses to hypoxia

Five min of continuous hypoxia was tolerated by all subjects. One of the MSNA recordings was lost during hypoxia in an older woman. Hypoxia decreased arterial O_2_ saturation with no differences between groups (young men: −18 ± 2%, young women: −15 ± 1%, older women: −15 ± 2%, *P* = 0.4031). This hypoxia protocol was poikilocapnic and decreased CO_2_ slightly (young men: −3 ± 0 mmHg, young women: −3 ± 1 mmHg, older women: −4 ± 1 mmHg, *P* = 0.5977 between groups). Neural and hemodynamic responses to continuous hypoxia are shown in Figure 1. MSNA responses to hypoxia were not different between groups (*P* = 0.9689). Hypoxia did not significantly alter mean BP in any group and there were no differences in responses between groups (*P* = 0.3750). Hypoxia increased HR in all groups and there was a trend toward an attenuated HR response to hypoxia in older women but this did not reach statistical significance (*P* = 0.1116). Hypoxia‐induced femoral artery vasodilation in all groups but this response was attenuated in young women compared to young men (*P* = 0.0330). There were no significant group differences in the femoral vascular conductance or resistance responses to hypoxia (Fig. 2).

## Discussion

### Summary and main findings

The main finding of this study is that young women have paradoxical femoral artery vasodilation to CPT despite similar increases in MSNA compared to young men. This finding supports a growing body of literature suggesting that young women have attenuated sympathetically mediated vasoconstriction. We also observed that femoral artery vasodilation to continuous hypoxia is not different in young men and young women but attenuated in older women. These data show that the physiological response to hypoxic stress may not be affected by sex but supports previous data that hypoxia‐mediated vasodilation is attenuated by aging.

### Vasodilatory response to the cold pressor test in young women

While it is largely accepted that CPT produces vasoconstriction, we observed that young women have paradoxical femoral artery vasodilation to CPT. We believe that this finding was overlooked in previous experiments since most studies examining vascular responses to CPT have (1) grouped male and female subjects together and (2) investigated forearm vascular responses where sex differences are less likely to occur. One previous study by Hogarth et al. found that women have an attenuated rise in calf vascular conductance to CPT despite a similar rise in MSNA compared to men (Hogarth et al. 2007). However, the average age of women in this study was 43 years and it is possible that if they had only included young, premenopausal women the observed sex differences would have been greater. While, blood flow responses to CPT were greater in young women, MSNA responses were similar which may indicate sex differences in vascular transduction. Mechanisms behind the change in transduction of sympathetic activity to vascular responses will be discussed further.

In contrast to previous studies (Jarvis et al. 2011) found blunted blood pressure responses to CPT in young women, in this study we observed that blood pressure responses to CPT were not different between groups. The reason for this opposing finding is unclear.

### Sex differences in the physiological responses to hypoxia

Previous studies support the concept that hypoxia‐induced vasodilation is attenuated with age (Kirby et al. 2009; Casey et al. 2011; Pollock et al. 2014); however, there were very few women in these studies and sex differences were not analyzed. In this study we observe that hypoxia‐induced vasodilation is attenuated in older women. A few studies have investigated sex differences to continuous hypoxemic stress with conflicting findings. Casey et al. (2014) performed isocapnic hypoxia to a target arterial O_2_ and found that hypoxia‐induced forearm vasodilation was greater in young women in early follicular phase compared to young men. Usselman et al. (2015) performed continuous hypoxia via rebreathing in men and women and observed similar changes in total peripheral resistance in young men and young women regardless of menstrual cycle phase. In the current study we also did not observe sex differences in hypoxia‐induced vasodilation similar to Usselman et al. These varying results could be due to potential differences in CO_2_ or tissue O_2_ which were controlled in the study by Casey et al. (2014). Since we did not control for tissue O_2,_ the metabolic demands of the tissue may have been different between groups, which is the primary regulator of hypoxia‐induced vasodilation.

Usselman et al. (2015) also reported greater sympathetic responses to hypoxic stress in young women during early follicular phase compared with young men. In the current study, we observed that increases in MSNA were not different between groups perhaps because we did not control for menstrual cycle phase in young women.

### Evidence of decreased sympathetic vasoconstrictor tone in young women

Our study adds to the emerging field of sex differences in vascular transduction. Studies in healthy humans suggest that MSNA is correlated to peripheral vasoconstriction in men but not in young women. Studies by Hart et al. (2009) showed that MSNA was only correlated with total peripheral resistance in young men and there was no correlation with young women. In a successive study, they found that *β*‐adrenergic receptor blockade with propranolol increased the relationship between MSNA and total peripheral resistance in young women (Hart et al. 2011). Another study by Kneale et al. (2000) found that intra‐arterial infusion of norepinephrine induced vasoconstriction in young men and induced vasodilation or blunted vasoconstriction in young women; pretreatment with propranolol significantly increased vasoconstriction in young women and had minimal effects in young men. Collectively, these studies suggest that young women have enhanced *β*‐adrenergic mediated vasodilation that dissociates the relationship between MSNA and vascular resistance. Therefore, given the same level of sympathetic activity (MSNA) and norepinephrine release from sympathetic nerve terminals, young women may have less vasoconstriction or vascular transduction due to enhanced norepinephrine binding to *β*‐adrenergic receptors. This study supports this notion since young women had paradoxical vasodilation to CPT, a stressor that increases MSNA and norepinephrine release. This phenomenon may be due to enhanced *β*‐adrenergic receptor stimulation by norepinephrine. It is also possible that *α*‐adrenergic receptor mediated vasoconstriction is impaired in young women but there are less previous studies to support this theory. Further experiments are needed to confirm the mechanism behind the vasodilatory response to CPT observed in young women.

This study adds to the body of literature of sex differences in peripheral blood flow responses to physiological stressors. Previous studies have found that blood flow responses to orthostatic and mental stress are enhanced in young women (Shoemaker et al. 2001; Yang et al. 2012, 2013). A previous study from our group found attenuated vasoconstriction to voluntary end‐expiratory apnea in young women compared to young men (Patel et al. 2014). This study adds to existing literature by demonstrating that young women have attenuated vasoconstriction and enhanced vasodilation to CPT. The mechanisms underlying the sex differences in responses to physiological stressors are yet to be determined.

### Limitations

While most of the young women in this study were taking hormonal contraception, this may not have affected our results. There are few studies on the effects of hormonal contraception on cardiovascular autonomic function. However, a recent study by Harvey et al. (2015) suggests that hormonal contraceptives do not alter MSNA or hemodynamics in young women.

## Conclusion

For the first time we show that young women have paradoxical vasodilation to the cold pressor test. This supports an emerging body of literature that suggests that young women have attenuated vasoconstriction or enhanced vasodilation to physiological stressors. Further studies are needed to elucidate the mechanisms behind the apparent sex differences to physiologic stressors.

## Conflict of Interest

On behalf of all authors, the corresponding author states that there is no conflict of interest.
